# On labyrinthine function loss, motion sickness immunity, and velocity storage

**DOI:** 10.3389/fneur.2024.1426213

**Published:** 2024-06-28

**Authors:** Jun Maruta

**Affiliations:** ^1^Department of Neurology, Icahn School of Medicine at Mount Sinai, New York, NY, United States; ^2^Department of Rehabilitation and Human Performance, Icahn School of Medicine at Mount Sinai, New York, NY, United States

**Keywords:** cerebellar nodulus, mal de débarquement, nystagmus, semicircular canals, space flight, spatial orientation, vestibular habituation, vestibulo-ocular reflex (VOR)

## Introduction

The space race set in motion by the Cold War intensified in the 1960s, with the Soviet Union first paving the path to manned space flight in 1961 (1). In high relevance to astronauts' mission readiness (2–5), experiments conducted in this decade, contrasting persons with functioning and bilaterally non-functioning labyrinths, solidified the contributions of the vestibular inputs to production of spatial perception and motion sickness during exposure to passive motion or microgravity (6). However, all forms of labyrinthine deactivation are not created equal. For example, patients may undergo total vestibular nerve section as a treatment for vertigo due to eighth nerve neuroma or Ménière's disease (7, 8); others may for varying causes sustain selective damage within the vestibular end-organs while retaining functioning nerves, as may be the case for candidates of vestibular implants (9, 10). Selective inactivation of otolithic signals may even be induced environmentally under microgravity in space. Examining information processing in the central vestibular mechanism known as velocity storage in various contexts of labyrinthine inactivation may shed further light on health and perceptual anomalies during or following space flight.

## What is velocity storage?

Activated by head rotation, large-field visual motion, or proprioceptive cues for continuous rotation, velocity storage is a central neural circuit that sustains a rotational velocity estimate of ongoing self-motion (11–16). The mechanism serves a working memory-like function of self-motion and spatial orientation to shape ocular and postural reflexes in the brainstem and presumably perception in the cerebral cortex (13–15, 17–19).

**Figure 1:**
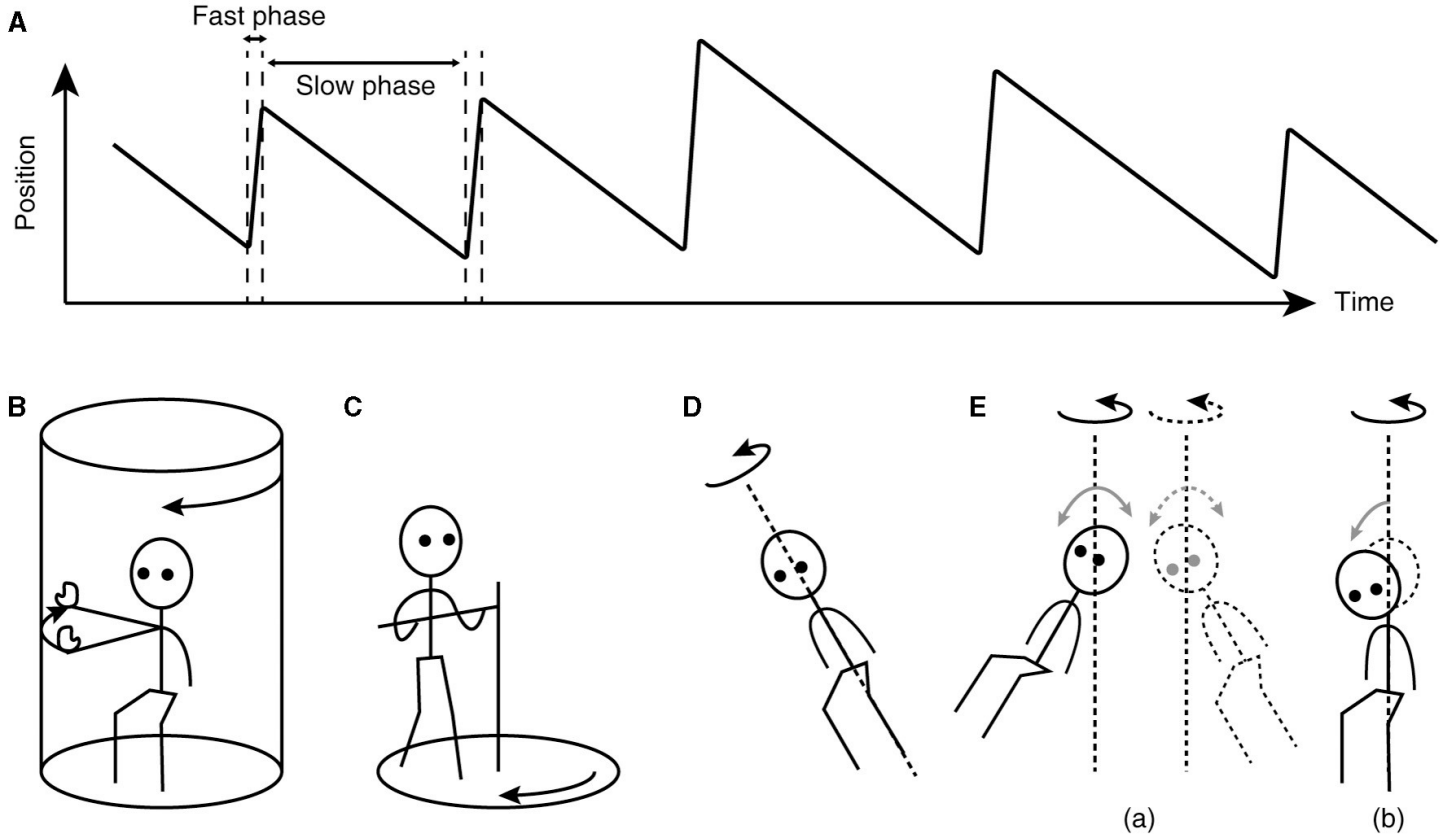
Characteristics of nystagmus and examples of unusual stimuli that can induce nystagmus. **(A)** Sawtooth pattern of eye position change over time during nystagmus. Slow-phase movements are directed against, thus in compensation for, the detected head rotation, whereas fast-phase movements facilitate maintenance of eye position within the oculomotor range. **(B)** Arthrokinetic stimulation. **(C)** Circular treadmill locomotion. **(D)** OVAR. **(E)** Whole-body sinusoidal PWR (a). As a variation in human testing, a disorienting sensation and motion sickness may be induced with a discrete, rather than continuous, head tilt while seated in a rotating chair (b). Note that in **(B, C)**, the head stays spatially stationary, and when conducted in darkness, the stimuli can generate nystagmus without labyrinthine or visual input. In **(D, E)**, at steady state, the semicircular canals do not signal the rotation continuing about the axis indicated by the dotted line.

The key to understanding the working of velocity storage has been in eye movement measurement. Nystagmus, such as occurs during the vestibulo-ocular reflex (VOR), is an automatic eye movement made in response to signals that indicate self-rotation relative to the surrounding world. Nystagmus facilitates the acquisition of visual information with the eyes moving in opposition to and in compensation for the detected rotation (slow phases) but for quick “resetting” interruptions (fast phases; Figure 1A). The presence of a common, sub-cerebral cortical mechanism for multimodal sensory integration had long been presumed based on experimental observations such as: (a) coordinated shaping, rather than a simple superposition, of nystagmus of different sensory origins takes place during combined stimulation (11); (b) the nystagmic reaction to either visual (optokinetic) or vestibular stimulation is subject to a “central inertia,” demonstrated with a prolonged response or after-response (11); (c) optokinetic nystagmus (OKN) can be induced in animals with visual cortical lesions (11, 20); and (d) bilateral vestibular nerve sections degrade OKN and abolish optokinetic after-nystagmus (OKAN) (21, 22).

Animal experiments in the 1970s demonstrated that spiking activity in certain second-order vestibular neurons correlated with the strength of either vestibularly- or optokinetically-induced nystagmus (23–25). With this discovery, the functional operation of the central processing system that soon came to be known as velocity storage became a valuable focus of rigorous model-based testing of behavioral and physiological data (13–15, 26, 27), These neurons belong to the vestibular-only (VO) class, so-termed to differentiate from other secondary vestibular neurons that display saccade or eye position sensitivity (28). Importantly, while the decay time constant of the primary afferent activity after the cessation of head angular acceleration, contributed by the semicircular canal endolymph's inertia, amounts only to 3–6 s, the activity of VO neurons is typically characterized with a time constant of 10–40 s, corresponding to the aforementioned “central inertia” (25, 29–32).

Besides vestibular or optokinetic means, and indeed in the absence of actual motion or visual motion, an illusion of body rotation (circular vection) and corresponding compensatory nystagmus can be induced in a stationary person when a rotating surround is passively followed with an extended arm (Figure 1B) (12) or when the person steps along a fictitious, circular trajectory on a counter-rotating floor (Figure 1C) (16). Compensatory nystagmus is also generated in the absence of vision or steady activation of coplanar semicircular canals when the subject is continuously rotated about a tilted axis (off-vertical axis rotation—OVAR; Figure 1D) (33–35) or when sinusoidally pitched or rolled during earth-vertical axis rotation (pitch/roll while rotating—PWR/RWR; Figure 1E) (36, 37).

That nystagmus can be generated and sustained without coplanar optokinetic or semicircular canal activation indicates that the velocity storage mechanism actively reconstitutes self-motion signals from multimodal sensory inputs. Additionally, the stored estimate of self-motion can be dynamically transformed such that the outcome nystagmus tends to align to the gravito-inertial field. For example, horizontal OKN induced while tilted laterally gives way to OKAN with a vertical component (38–40). Similarly, the per-rotatory nystagmus in response to off-center rotation with forward or backward tangential motion develops an out-of-plane, vertical component as the centripetal acceleration tilts the gravito-inertial field (41). Thus, velocity storage is also equipped with orienting properties to act as a “neural gyroscope” (40, 42, 43), making it more fitting to be identified as “working memory-like” rather than mere storage.

The properties of velocity storage are nearly exclusively characterized by the dynamics of slow-phase eye velocity of nystagmus. However, many of so-characterized properties have also been demonstrated in the activity of VO neurons (29, 44, 45). Insight into the working of velocity storage has been conversely derived from VO neuron activity as well. For example, while most VO neurons receive convergent inputs from different semicircular canal and otolith afferents, many show preferences to activation near or orthogonal to the plane of a specific push-pull canal pair (29, 46). Thus, VO neurons appear to collectively code the three-dimensional action of velocity storage using a coordinate system consistent with the geometric alignment of the semicircular canals and extraocular muscles, which is common across lateral- or frontal-eyed species (29, 47). Further, while vestibular afferents cannot differentiate active from passive motion, VO neurons reportedly show different responses (48–51). Accordingly, velocity storage may be activated and controlled differently during active and passive motion.

Velocity storage is under the inhibitory control of the cerebellar nodulus and, likely, the adjacent caudal uvula. Damage to these areas destabilizes velocity storage, triggering periodic alternating nystagmus and elongating the time constant of the decay of the VOR nystagmus during a rotational test (52–55). Such damage also results in loss of compensatory nystagmus during OVAR and sinusoidal PWR/RWR (56, 57) as well as in a compromised ability to reorient eye velocity to the gravito-inertial field (55, 57, 58). Reversible unilateral inactivation of the nodulus induces spontaneous nystagmus in darkness with contralaterally-directed slow phases and abnormal eye velocity orientation to gravity (59). The nodulus and caudal uvula target a wide variety of cell types in the vestibular nuclei, including VO neurons (60). Nodular micro-electrical stimulation results in shortening of the decay time constant of the VOR or OKAN with ipsilateral slow phases (60, 61), but may also yield further different effects depending on the duration and exact location of the stimulation (59, 62).

Lastly, velocity storage is malleable. For example, repeated rotation in darkness shortens the VOR decay time constant (decreased “central inertia”) in a long-retained effect known as vestibular habituation (63–65). The spatial orientation properties of velocity storage, such as observed in eye movements outside the stimulus plane during the VOR, can also be modified (66, 67), possibly to facilitate adaptation to a new gravito-inertial environment (68–70). Relatedly, the spatial tuning of VO neurons can be modified with prolonged tilt (71). As the cerebellum plays a major role in motor learning and calibration (72, 73), the malleability of velocity storage likely depends on the nodulus and uvula (64). Mal de débarquement syndrome, a chronic illness primarily characterized by a persistent illusory perception of self-motion, is thought to result from a failure in velocity storage to readapt to a normal acceleration environment after adapting to passive motion (74–76).

## Correlates of velocity storage perturbation

Studies conducted in humans and animals with bilaterally non-functioning labyrinths, including those from the 1960s, found that these subjects were immune to motion sickness when exposed to otherwise highly provocative vestibular tests, such as PWR/RWR, OVAR, and other unusual motion stimuli (6, 77–79). Such immunity merits re-examination in parallel with patterns of velocity storage activation (Table 1). Differences and similarities in perceptual experiences are also considered when illuminating information is available.

**Table 1:** Exemplar effects of localized vestibular-related functional inactivation.

**Cause of vestibular-related functional inactivation**	**Effect on velocity storage**	**Effect on motion sickness susceptibility**
Non-surgical loss of bilateral labyrinthine function	•Loss or significant reduction of OKAN (80–83)	•Immunity for physical motion (6, 78) •Immunity for visual motion (84)
Bilateral vestibular neurectomy	•*Loss of OKAN* (21, 22, 35)	•No change when physical motion is combined with visual motion (7)
Canal plugging	•*Loss of ability to generate compensatory nystagmus during sinusoidal PWR/RWR* (36, 85)	•*Reduction for motion generated on a swing with head unrestrained* (86)
Utriculosacculectomy	•*Reduced time constant* (87) •*Loss of ability to generate compensatory nystagmus during OVAR* (88)	•*Immunity for movement in a rotating environment* (89) •*Reduction for sinusoidal pitching with conflicting visual stimulus* (90)
Exposure to microgravity	•Reduced time constant (91–93) •During prolonged exposure, possible restoration of time constant (93)	•Reduction for PWR/RWR (3, 94, 95) •Increase for head movements with eyes open (3, 96)
Nodulo-uvular lesion	•*Increased time constant* (54, 55, 64) •*Loss of spatial orientation properties* (55, 57, 58) •*Loss of ability to generate compensatory nystagmus during sinusoidal PWR/RWR and OVAR* (56, 57)	•*Immunity for motion generated on a swing* (97, 98) •*No change in “pica” behavior for hypergravity stimulation* (99) •*Acutely, vomiting* (55, 100) •Acutely, vomiting (52, 53)

Italicized entries indicate observations based on animal experiments.

Vestibular habituation, identified with a shortened velocity storage time constant, reportedly reduces susceptibility to motion sickness (101, 102). The GABA-B agonist baclofen has been indicated to also reduce the velocity storage time constant and motion sickness susceptibility to RWR, but reversibly (103, 104). Given that bilateral vestibular nerve sections result in loss of OKAN (21, 22), and that loss of or impaired vestibular functions due to ototoxicity or other causes significantly reduces OKAN (80–83), the immunity to motion sickness to provocative motion stimuli in labyrinthine-defective individuals may be explained by their loss of or reduced ability to store velocity signals. However, motion sickness and circular vection can still be induced with head movements in a rotating visual environment after vestibular neurectomy (7). The effect of baclofen on visually induced motion sickness is not known.

Neuronally, while the ability to store velocity signals appears to depend on the wellbeing of the primary afferents, there is no evidence that VO neurons undergo anterograde transneuronal degeneration after vestibular nerve sections (105, 106). Neuronal activities ascribed to velocity storage functions that survive labyrinthine deactivation remain to be identified. Behaviorally however, nystagmus and circular vection can be induced in labyrinthine-defective individuals during fictitious circular stepping around in darkness (Figure 1C) (16), providing strong evidence that reconstitution of self-motion velocity signals and their storage are separate processes.

Motion sickness is typically induced in a context of passive rather than active motion (107, 108). Likewise, VO neurons are reportedly more sensitive to passive than active head movements (48–51). Participation of these neurons in the vestibular-autonomic circuits is not known (109–113), but motion sickness susceptibility is likely not directly increased as a simple consequence of increased VO neuron activation. For example, while congruent optokinetic and vestibular inputs can synergistically activate VO neurons and incongruent ones antagonistically (25–27, 29, 114), vision of a stationary surround (i.e., optokinetic input congruent with physical motion) protects against motion sickness and perceptual disorientation during PWR/RWR or OVAR (79, 115–120). Curiously, under microgravity in space or during parabolic flight, active head movements with vision of a stationary surround is more provocative than the same movements without vision (3, 96).

Also curiously, while PWR/RWR is highly provocative of motion sickness and perceptually disorienting on earth, it is not so under microgravity in space or during parabolic flight (3, 94, 95). Mirroring this environmental inactivation of otolithic signals, after bilateral utricular nerve section and saccular macula destruction, previously susceptible squirrel monkeys reportedly become immune to motion sickness while unrestrained inside a rotating cage (89), a condition that likely generates provocativeness through a PWR/RWR-like mechanism. By contrast, semicircular canal deactivation precipitated by ototoxicity, sparing otolithic functions, has also been shown to induce immunity to motion sickness in unrestrained squirrel monkeys in a rotating cage (121). Thus, the provocativeness of PWR/RWR appears to arise from central integration of both the semicircular canal and otolithic signals. By parallel, the ability to generate nystagmus during sinusoidal PWR/RWR is lost in macaque monkeys after selective deactivation of the semicircular canal function with canal plugging (36, 85). The counterpart effect of selective deactivation of the otolithic input does not seem to have been experimentally tested, but nystagmus generation during sinusoidal PWR/RWR is also thought to require both otolithic and semicircular canal inputs (57, 67, 85).

The involvement of the cerebellar nodulus and uvula provides another parallel between velocity storage and motion sickness. The nodulo-uvular involvement in motion sickness production has long been suspected based on observations that nodulo-uvular lesions rendered experimental animals immune to motion sickness (97, 98). Doubts raised against this view (99, 100) may be partially addressed by considering the acute vs. chronic effects of such lesions and the presence of parallel vestibular-cerebellar circuits that can produce counteracting autonomic effects (52, 53, 111, 113, 122, 123). It is presumably within such complex neural interactions that learning takes place so that evoked symptoms of motion sickness and future susceptibility diminish even when the provocative situation is unchanged (4, 70). Motion sickness is said to be most severe when the orientation and autonomic regulation systems are undergoing rapid recalibration (4). Whether understanding the neural basis of velocity storage malleability improves the predictability of motion sickness susceptibility to specific stimuli or situations remains to be seen. The physiology of and the circuitries that control and are controlled by VO neurons likely serve a focal point of future studies (19, 113), an approach thus far possible chiefly in animal-based experiments.

## Conclusion

Clues from various forms of labyrinthine and central loss of vestibular function connect spatial perception, motion sickness, and velocity storage as parallel phenomena. Similar partially overlapping parallelisms from different perspectives have previously been suggested (19, 113). A possible pitfall of such thinking is that these phenomena may be just that—parallel but unrelated. No matter, the paths to fill the knowledge gaps are largely unpaved and promise abundant scientific opportunities. Continuing development in space exploration and technology behooves us to advance the field.

## Author contributions

JM: Writing – original draft, Writing – review & editing.
